# Disparities in Medication Use for Criminal Justice System–Referred Opioid Use Disorder Treatment

**DOI:** 10.1001/jamahealthforum.2024.2807

**Published:** 2024-09-06

**Authors:** J. Travis Donahoe, Julie M. Donohue, Brendan K. Saloner

**Affiliations:** 1 Department of Health Policy and Management, University of Pittsburgh School of Public Health, Pittsburgh, Pennsylvania; 2 Department of Health Policy and Management, Johns Hopkins University Bloomberg School of Public Health, Baltimore, Maryland

## Abstract

**Importance:**

Individuals with opioid use disorder (OUD) and criminal justice system involvement experience high rates of overdose death. Historical data point to limited use of medications for opioid use disorder (MOUD) in criminal justice system–referred treatment for OUD as playing a role. However, how MOUD use among those referred to treatment by the criminal justice system has changed relative to other referral sources over time is still unclear, as well as how it varies across states.

**Objective:**

To examine disparities in the use of MOUD between individuals referred to treatment by the criminal justice system compared to other referral sources over time.

**Design, Setting, and Participants:**

This cross-sectional study included admissions to specialty substance use treatment facilities for OUD in the national Treatment Episodes Dataset–Admissions from 2014 to 2021. Logistic regression models were used to examine trends in the probability of MOUD use among individuals with and without criminal justice referrals for OUD treatment, as well as any differential trends by state. The data were analyzed from September 2023 to August 2024.

**Main Outcome and Measure:**

The main outcome was the probability that treatment for individuals with OUD included MOUD.

**Results:**

A total of 3 235 445 admissions were analyzed in the study data. Among individuals referred to OUD treatment by the criminal justice system, the probability that treatment included MOUD increased by 3.42 percentage points (pp) (95% CI, 3.37 pp to 3.47 pp) annually from 2014 to 2021. This was faster than the increase in the probability of MOUD use for noncriminal justice–referred admissions (2.49 pp [95% CI, 2.46 pp to 2.51 pp) and reduced, but did not eliminate, disparities in MOUD use between individuals with and without criminal justice system–referred treatment. In 2021, only 33.6% of individuals in criminal justice system–referred treatment received MOUD, 15.6 pp lower than for individuals referred to treatment by other sources. Trends in the probability of MOUD use varied substantially for individuals in criminal justice system–referred treatment across states, but very few experienced enough growth to eliminate this disparity.

**Conclusions and Relevance:**

The results of this cross-sectional study suggest that targeted efforts to address persistent disparities in MOUD use among those with OUD and criminal justice system involvement are needed to address the poor health outcomes experienced by this population.

## Key Points

**Question:**

Have disparities in medication for opioid use disorder (MOUD) treatment between individuals referred by the criminal justice system and other sources decreased?

**Findings:**

This cross-sectional study, analyzing more than 3 million admissions to treatment centers for opioid use disorder between 2014 and 2021, found that although the use of MOUD among people referred to treatment by the criminal justice system has increased, it remains far lower than for people referred by other sources. This disparity exists in most states and is closing slowly.

**Meaning:**

Targeted efforts to increase MOUD use among individuals with opioid use disorder and criminal justice system involvement are needed to address the poor outcomes experienced by this population.

## Teaser

This cross-sectional study analyzes the disparity in the use of medications for opioid use disorder between individuals referred to treatment by the criminal justice system compared to other referral sources over time.

## Introduction

Many people in the criminal justice system have opioid use disorder (OUD). In 2019, an estimated 14.5% of people in jails had OUD,^1^ 9 times more than in the general population.^2^ Furthermore, individuals with criminal justice system involvement experience a high risk for overdose death and other poor outcomes. Among people who are incarcerated, overdose mortality rates are significantly elevated after release from incarceration^3,4^ and community supervision.^5^ OUD among people with criminal justice system involvement also contributes to increased rates of recidivism and reincarceration.^6^

One potential contributor to these poor outcomes is the limited use of medication for opioid use disorder (MOUD) in criminal justice settings. MOUD is the most effective treatment for reducing mortality from OUD,^7,8^ including for people with criminal justice system involvement.^9,10,11,12,13,14,15^ However, few jails^1^ and prisons^16^ offer MOUD. This partly reflects significant roadblocks to paying for MOUD in carceral settings, not the least of which is the federal inmate exclusion policy which requires states to terminate or suspend Medicaid coverage when someone is incarcerated.^17^ MOUD use has also historically been low in other criminal justice settings, such as drug courts^18^ and specialty treatment programs referred by the criminal justice system,^19^ that are explicitly intended to boost treatment rates for opioid and other substance use disorders. In 2014, just 5% of people referred to specialty treatment for OUD by the criminal justice system received MOUD compared to 41% of people referred to treatment by other sources.^19^

Many studies on MOUD use in criminal justice settings have focused on data prior to several state Medicaid expansions and regulatory changes during the COVID-19 pandemic, both of which increased MOUD use among people with criminal justice involvement.^20,21^ Moreover, a variety of national, state, and local initiatives have been promoting MOUD in criminal justice settings in recent years.^22,23^ These factors may have helped close the disparity in MOUD use for people referred to OUD treatment by the criminal justice system and other referral sources. However, to our knowledge, this has not yet been characterized in the literature.

This cross-sectional study uses national data on all opioid-related admissions to publicly funded substance use disorder treatment facilities to examine whether the disparity in MOUD use between individuals referred to OUD treatment by the criminal justice system and individuals referred to OUD treatment by other sources has closed over time. We also examine whether the disparity has closed differentially across states. This is important given the substantial heterogeneity in efforts to increase MOUD in criminal justice settings across states,^22,23^ which may have closed the disparity in some states but not others. These findings can help inform interventions at the federal, state, and local levels to increase the effectiveness of OUD treatment and improve outcomes in criminal justice settings.

## Methods

### Study Data and Population

We used data on all completed admissions to substance use disorder treatment facilities from the Treatment Episodes Dataset–Admissions (TEDS-A) from 2014 to 2021. We followed the Strengthening the Reporting of Observational Studies in Epidemiology (STROBE) reporting guideline. No institutional board review or informed consent waiver was required for this analysis of publicly available and deidentified data.

The types of facilities that report to TEDS-A vary across states; however, they typically include all facilities that receive state or federal funds (eg, block grants) and additional facilities as imposed by state-specific reporting requirements.^24^ We limited the sample to individuals 18 years and older who reported an opioid as the primary substance that they used. We excluded admissions with missing information for client demographics, socioeconomic characteristics, and the exposure and outcomes variables defined herein. Further, we excluded data from 7 states that did not report data in at least 1 year (Delaware, Idaho, Maryland, New Mexico, Oregon, South Carolina, and Washington), Puerto Rico, and 4 states that did not report any MOUD use or only 1 admission with MOUD, which suggested potential data quality problems (Oklahoma, Montana, Virginia, and West Virginia).^19^ We also conducted sensitivity using data from 2014 and 2021 alone and a larger number of states for which data was available in those years (eAppendix 2 in Supplement 1).

### Exposure and Outcome Variables

Exposure was defined as referral to treatment by the criminal justice system. This was constructed using the “court/criminal justice referral/Driving Under the Influence/Driving While Intoxicated” category of the “PSOURCE” variable in the TEDS-A data, which indicates treatment admissions where the referral source was “any police official, judge, prosecutor, probation officer or other person affiliated with a federal, state, or county judicial system.”^25^ This captures a wide variety of criminal justice system referral sources, including probation and parole officers, court-ordered treatment, and law enforcement–assisted diversion programs. Referrals by probation and parole officers also capture referrals made following release from jails and prisons, due to these officers typically handling postrelease placements. Comparison referrals included referrals by all other sources—individuals (oneself, family, and/or friends), health care professionals, schools, employers, and other community organizations.

The outcome was an indicator of whether any medication was included in a client’s treatment plan. We measured this using the “METHUSE” variable in the TEDS-A data,^25^ consistent with prior research.^19,21,26^ Facilities are instructed to report medication use for a treatment admission if any MOUD, including methadone, buprenorphine, or naltrexone, was included as part of the individual’s treatment plan.

### Statistical Analysis

The data were analyzed from September 2023 to August 2024. We fit logistic regression models that predicted the probability of MOUD use as a function of a binary variable for whether treatment was referred by the criminal justice system, a continuous variable for year, interactions between criminal justice referral and a continuous variable for year, and individual characteristics (age, sex, race and ethnicity, educational attainment, US Census region, and self-reported co-occurring harmful alcohol and benzodiazepine use). We controlled for race and ethnicity as there are well-known differences in receipt of medications to treat OUD^27^ and criminal justice involvement^28^ by race and ethnicity. We used the “RACE” and “ETHNIC” variables provided in the TEDS-A dataset.

The interaction term between criminal justice referrals and year allowed trends in the probability of MOUD to differ between individuals with criminal justice system referrals to treatment and other referral sources. For all analyses, we report results as average marginal effects^29^ and use robust standard errors. Using our estimates, we also calculated fitted probabilities that treatment admissions included MOUD separately under counterfactuals that all admissions were referred by the criminal justice system and were referred by other sources each year from 2014 to 2021.

We next examined whether disparities in MOUD use closed differentially for individuals in different states. To do this, we stratified the models by state. For each state, we quantified the trend in the probability of MOUD use for clients with criminal justice referrals to treatment. We also quantified a benchmark for each state, defined as the trend needed to achieve at least 50% probability of MOUD use among individuals referred to treatment by the criminal justice system in 2021. This is approximately the probability of MOUD use for noncriminal justice referrals in the TEDS-A data in 2021 and in other large population-based studies.^30^ Additional details are provided in eAppendix 1 in Supplement 1. Analyses were implemented in Stata statistical software, version 18 (StataCorp LLC). The results of 2-sided testing with *P* values less than .05 were considered statistically significant.

## Results

### Descriptive Statistics

The final analytic sample included data on 3 235 445 total OUD admissions across 40 states (eTable 1 in Supplement 1; Table 1). Overall, 509 765 treatment admissions (15.8%) were referred by the criminal justice system. Most admissions involved individuals aged 25 to 54 years (79.5%) and male individuals (63.5%). Heroin was the most common primary opioid used (76.8%), although the use of other opiates and synthetics increased in 2021 (eTable 2 in Supplement 1). The overall racial and ethnic composition of individuals was 15.3% Hispanic, 10.4% non-Hispanic Black, 1.1% non-Hispanic American Indian or Alaska Native, 69.4% non-Hispanic White, and 3.8% other race and ethnicity (non-Hispanic individuals whose race was Asian, Native Hawaiian, other Pacific Islander, another single race, 2 or more races, or missing from database).

**Table 1.** Sample Characteristics From Treatment Episodes Dataset–Admissions, 2014 to 2021

Characteristic	No. (%)			*P* value^a^
	All admissions	Noncriminal justice system referrals	Criminal justice system referrals	
Admissions	3 235 445 (100)	2 725 680 (84.2)	509 765 (15.8)	NA
Age, y				
18-24	412 435 (12.7)	330 238 (12.1)	82 197 (16.1)	<.001
25-34	1 392 215 (43.0)	1 146 387 (42.1)	245 828 (48.2)	<.001
35-54	1 180 040 (36.5)	1 016 824 (37.3)	163 216 (32.0)	<.001
≥55	250 755 (7.8)	232 231 (8.5)	18 524 (3.6)	<.001
Sex				
Female	1 180 212 (36.5)	1 007 666 (37.0)	172 546 (33.8)	<.001
Male	2 055 233 (63.5)	1 718 014 (63.0)	337 219 (66.2)	<.001
Race and ethnicity				
Hispanic	495 089 (15.3)	426 729 (15.7)	68 360 (13.4)	<.001
Non-Hispanic Black	337 034 (10.4)	295 861 (10.9)	41 173 (8.1)	<.001
Non-Hispanic American Indian or Alaska Native	36 900 (1.1)	29 752 (1.1)	7148 (1.4)	<.001
Non-Hispanic White	2 244 713 (69.4)	1 866 400 (68.5)	378 313 (74.2)	<.001
Other race and ethnicity^b^	121 709 (3.8)	106 938 (3.9)	14 771 (2.9)	<.001
Educational attainment				
<High school	796 224 (24.6)	664 700 (24.4)	131 524 (25.8)	<.001
High school graduate	2 301 636 (71.1)	1 938 728 (71.1)	362 908 (71.2)	.02
College graduate	137 585 (4.3)	122 252 (4.5)	15 333 (3.0)	<.001
Primary opioid used				
Heroin	2 484 178 (76.8)	2 116 300 (77.6)	367 878 (72.2)	<.001
Nonprescription methadone	18 653 (0.6)	16 433 (0.6)	2220 (0.4)	<.001
Other opiates and synthetics	732 614 (22.6)	592 947 (21.8)	139 667 (27.4)	<.001
Co-occurring substance use				
Harmful alcohol use	465 532 (14.4)	387 311 (14.2)	78 221 (15.3)	<.001
Harmful benzodiazepine use	326 117 (10.1)	288 362 (10.6)	37 755 (7.4)	<.001
Census region				
Northeast	1 625 370 (50.2)	1 403 962 (51.5)	221 408 (43.4)	<.001
Midwest	660 967 (20.4)	536 468 (19.7)	124 499 (24.4)	<.001
South	438 165 (13.5)	357 457 (13.1)	80 708 (15.8)	<.001
West	510 943 (15.8)	427 793 (15.7)	83 150 (16.3)	<.001

Abbreviation: NA, not applicable.

^a^ *P* values are from a 2-sample *t* test for differences between individuals with criminal justice system referrals and referrals from other sources.

^b^ The other race and ethnicity category includes non-Hispanic individuals whose race was Asian, Native Hawaiian, other Pacific Islander, another single race, 2 or more races, or missing in the Treatment Episodes Dataset–Admissions data.

Table 1 also reports descriptive statistics stratified by referral source. Relative to individuals referred to treatment by other sources, criminal justice–referred individuals were younger, more likely to be male and non-Hispanic White, and more likely to live in the Midwest, South, or West Census regions.

The most common sources of criminal justice treatment referrals were probation and parole officers (26.6%) (eTable 3 in Supplement 1). This was followed by state and federal courts (16.6%), diversion programs (6.4%), other courts (6.2%), other legal entities (4.6%), prison (3.6%), and driving under the influence/driving while intoxicated programs (1.3%). The specific entity was recorded as other (no specific entity recorded) for 7.8% of criminal justice referrals and missing for 26.8% of criminal justice referrals.

### Trends in MOUD Use for Individuals Referred to Treatment by the Criminal Justice System

Results from the regression models are presented in Table 2. On average, individuals referred to treatment by the criminal justice system were 21.06 percentage points (pp) (95% CI, −21.19 pp to −20.94 pp) less likely to receive MOUD as part of treatment compared to individuals who were referred to treatment by other sources. Overall (ie, across both criminal justice–referred and noncriminal justice–referred admissions), the probability that treatment admissions included MOUD increased over time by 2.61 pp (95% CI, 2.58 pp to 2.63 pp) annually. Compared to individuals referred to treatment by other sources, the probability that treatment admissions included MOUD increased by 0.94 pp (95% CI, 0.88 pp to 0.99 pp) more each year for individuals referred to treatment by the criminal justice system.

**Table 2.** Logistic Regression Model of the Probability of Treatment With Medication for Opioid Use Disorder (MOUD)^a^

Variable	Mean marginal effect (robust SE) [95% CI], pp	*P* value
Referred by criminal justice system	−21.06 (0.06) [−21.19 to −20.94]	<.001
Year	2.61 (0.01) [2.58 to 2.63]	<.001
Criminal justice system referred × year	0.94 (0.03) [0.88 to 0.99]	<.001
Age (reference, 18-24 y), y		
25-34 y	6.54 (0.08) [6.38 to 6.69]	<.001
35-54 y	12.51 (0.08) [12.35 to 12.67]	<.001
≥55	21.57 (0.12) [21.34 to 21.80]	<.001
Female sex	5.34 (0.05) [5.24 to 5.45]	<.001
Race and ethnicity (reference, non-Hispanic White)		
Hispanic	−0.48 (0.07) [−0.62 to −0.33]	<.001
Non-Hispanic American Indian or Alaska Native	5.07 (0.24) [4.59 to 5.55]	<.001
Non-Hispanic Black	−2.86 (0.09) [−3.03 to −2.69]	<.001
Other race and ethnicity^b^	−1.77 (0.13) [−2.03 to −1.51]	<.001
Education (reference, <high school)		
High school graduate	−1.99 (0.06) [−2.10 to −1.87]	<.001
College graduate	−4.71 (0.13) [−4.97 to −4.46]	<.001
Primary opioid used (reference, heroin)		
Nonprescription methadone	14.30 (0.35) [13.62 to 14.98]	<.001
Other opiates and synthetics	−0.53 (0.06) [−0.66 to −0.41]	<.001
Reported harmful alcohol use	−10.24 (0.07) [−10.38 to −10.10]	<.001
Reported harmful benzodiazepine use	−6.75 (0.08) [−6.91 to −6.58]	<.001
Census region (reference, Northeast)		
Midwest	−3.60 (0.07) [−3.74 to −3.46]	<.001
South	−24.46 (0.07) [−24.60 to −24.33]	<.001
West	10.28 (0.08) [10.13 to 10.43]	<.001
Unadjusted probability of MOUD use, mean (SD)	36.45 (0.48)	NA

Abbreviations: NA, not applicable; pp, percentage point; SE, standard error.

^a^ Data are from the Treatment Episodes Dataset–Admissions, 2014-2021, which included a total of 3 235 445 admissions. The table reports the probability that admissions to specialty substance use treatment facilities involved the use of medication for opioid use disorder (ie, buprenorphine, methadone, and naltrexone) on each variable in the table.

^b^ The other race and ethnicity category includes non-Hispanic individuals whose race was Asian, Native Hawaiian, other Pacific Islander, another single race, 2 or more races, or missing in the Treatment Episodes Dataset–Admissions data.

**Figure 1.**
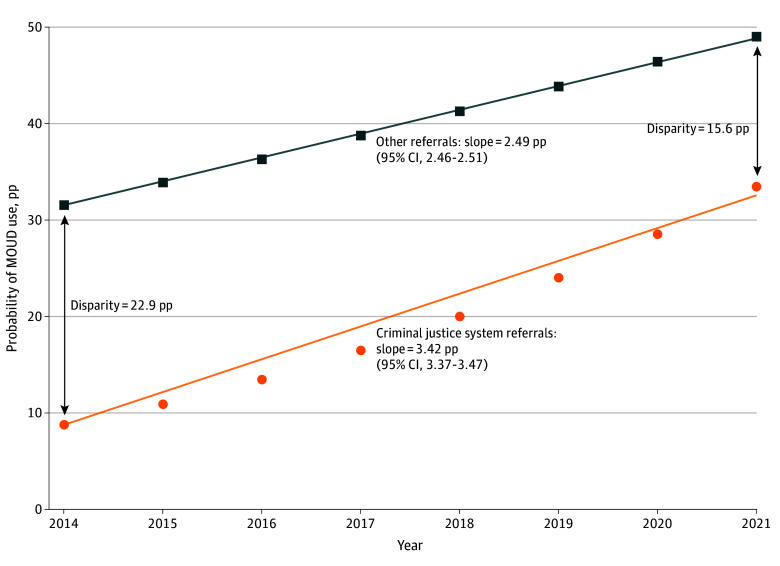
Trends in Medication for Opioid Use Disorder (MOUD) Use for Individuals Referred by Criminal Justice System and Other Sources The data are from the Treatment Episodes Dataset–Admissions, 2014 to 2021. The point estimates denote the probability that a treatment admission included MOUD in each year, separately for clients referred to treatment by the criminal justice system (depicted as orange circles) and other sources (depicted as dark blue squares), adjusted for patient characteristics. The figure overlays trend lines for each type of treatment referral, and the slope (95% CI) values are provided below each line. The disparities in the probability of MOUD use for individuals referred to treatment by the criminal justice system compared to other sources (holding individual-level characteristics fixed) are reported for 2014 and 2021. Percentage points are abbreviated as pp. See eAppendix 1 in Supplement 1 for more detail.

Based on the regression estimates, Figure 1 shows fitted probabilities that treatment admissions included MOUD each year, separately assuming that all admissions were referred to treatment by the criminal justice system and that all admissions were referred by other sources (eTable 4 in Supplement 1 for numerical results). The figure also overlays the trend in the probability of MOUD use for individuals referred to treatment by the criminal justice system (3.42 pp [95% CI, 2.46 pp to 2.51 pp]) and for individuals referred to treatment by other sources (2.49 pp [95% CI, 2.46 pp to 2.51 pp]). The differentially greater rate of increase in the probability of MOUD use for individuals referred to treatment by the criminal justice system with respect to time helped reduce the disparity in the probability of MOUD use between individuals referred to treatment by the criminal justice system and other referral sources (from 22.9 pp in 2014 to 15.6 pp in 2021).

A significant disparity in MOUD use between individuals referred to treatment by the criminal justice system and other referral sources remained in 2021. Overall, 49.3% of individuals referred to OUD treatment by noncriminal justice sources received MOUD. A total of 33.6% of individuals referred to OUD treatment by criminal justice sources received the same treatment, adjusting for individual characteristics. Results from the analysis with additional states are reported in eTable 5 and eFigure 1 in Supplement 1 and are very similar.

### Changes in Disparities in MOUD Use by State

**Figure 2.**
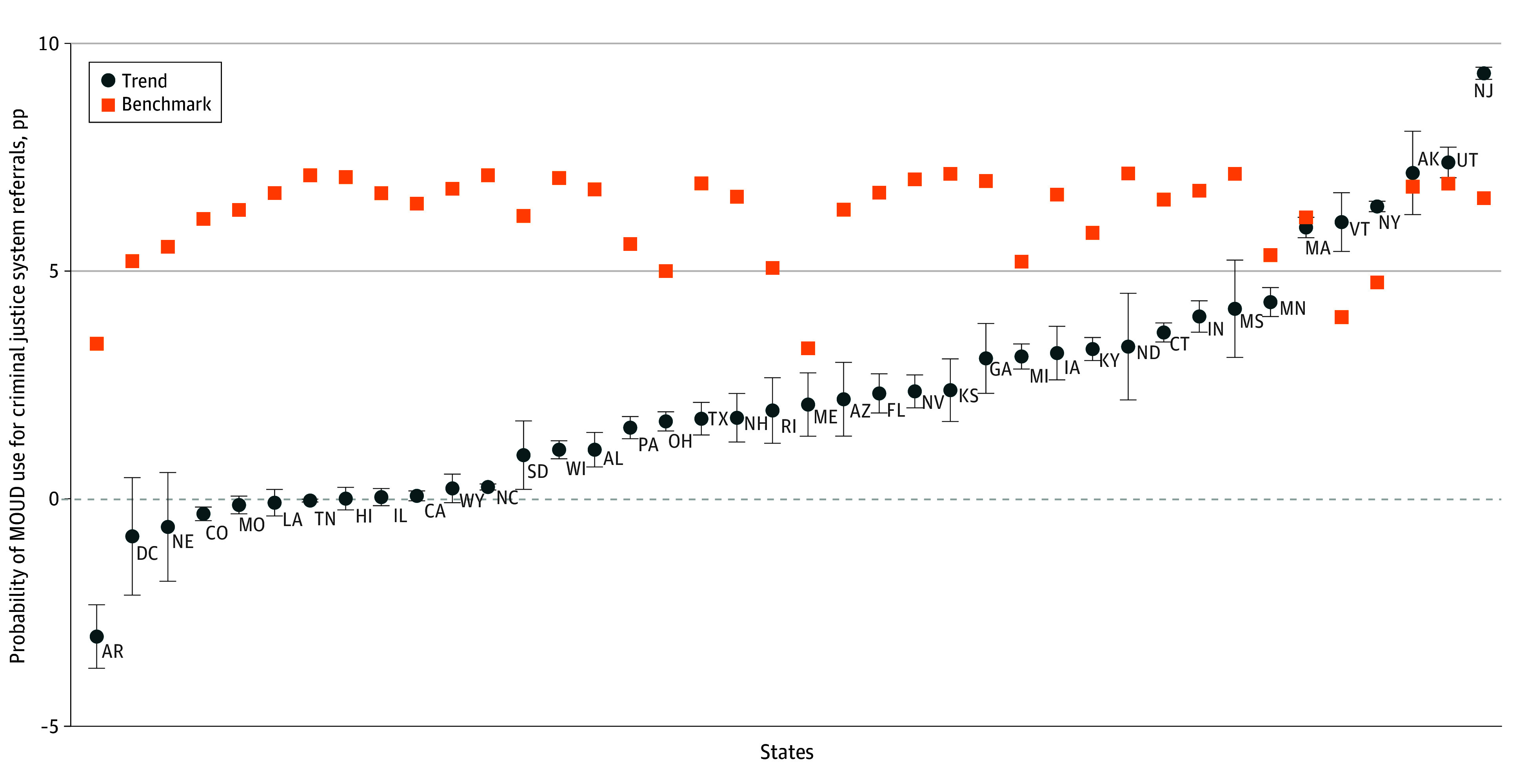
Trends in Medication for Opioid Use Disorder (MOUD) Use for Individuals Referred by the Criminal Justice System by State The data are from the Treatment Episodes Dataset–Admissions, 2014 to 2021. The figure shows the annual rate of growth in MOUD use for clients referred to OUD treatment by the criminal justice system in different states. Growth rates and 95% CIs, which are represented by the error bars, were estimated using logistic regression of the probability of MOUD use on an indicator, including whether treatment was referred by the criminal justice system, year, an interaction between whether treatment was referred by the criminal justice system and year, and client characteristics, stratified by state. The figure also shows a benchmark for each state, defined as the rate of growth needed for 50% of individuals referred to treatment by the criminal justice system in each state to receive MOUD in 2021. Percentage points are abbreviated as pp. See eAppendix 1 in Supplement 1 for more detail and eTable 5 in Supplement 1 for numerical results.

Figure 2 reports results from the analysis of the disparity in MOUD use for individuals referred to treatment by the criminal justice system in different states (eTables 6 and 7 in Supplement 1). The figure shows 2 estimates. First, it shows the trend in the probability of MOUD use for individuals referred to treatment by the criminal justice system in each state from 2014 to 2021. Second, it shows a benchmark for what the trend would have needed to be for at least half of criminal justice system–referred treatment admissions to have received MOUD in 2021. Variability in the benchmark across states is driven by baseline variability in probability of MOUD use among criminal justice system–referred treatment admissions.

A substantial amount of heterogeneity was found in trends in MOUD use among criminal justice system–referred treatment across states. Several states experienced negative or no (ie, not statistically significantly different than zero) growth in the probability of MOUD use for criminal justice system–referred treatment. These included Arkansas, the District of Colombia, Nebraska, Colorado, Montana, Louisiana, Tennessee, Hawaii, Illinois, California, and Wyoming. Most states experienced increased MOUD use for criminal justice system–referred treatment, but not enough to eliminate disparities. These included South Dakota, Wisconsin, Alabama, Pennsylvania, Ohio, Texas, New Hampshire, Rhode Island, Maine, Arizona, Florida, Nevada, Kansas, Georgia, Michigan, Iowa, Kentucky, North Dakota, Connecticut, Indiana, Mississippi, and Minnesota. Lastly, a small number of leading states experienced sufficient growth to close the disparity. These included Massachusetts, Vermont, New York, Alaska, Utah, and New Jersey.

## Discussion

Consistent with prior research,^19^ this cross-sectional study, which included updated data from the past decade, documented low use of MOUD for OUD treatment among individuals referred by the criminal justice system. On average, clients referred to OUD treatment by the criminal justice system were 21 pp (58%) less likely to receive MOUD during treatment compared to individuals who were referred to treatment by other sources. We found that the use of MOUD in criminal justice system–referred treatment has increased substantially since 2014, helping reduce this disparity. Although fewer than 10% of clients referred to treatment by the criminal justice system received MOUD in 2014,^19^ close to one-third did in 2021.

However, while increased use of MOUD in criminal justice settings is encouraging, use of MOUD is still far lower than is likely needed based on comparisons with MOUD use among individuals who are referred to OUD treatment by other sources and have similar characteristics. In 2021, individuals referred to treatment by the criminal justice system remained 15.6 pp less likely to receive MOUD compared to similar individuals referred to treatment by other sources. This is concerning due to MOUD being the most effective way to reduce overdose mortality among people with OUD^7^ and the very high rate of overdose death among people with criminal justice system involvement.^31^

To shed additional light on this issue, we examined whether it closed differentially across states. We found that the disparity was highly persistent across states and that, in total, just 6 states (Massachusetts, Vermont, New York, Arkansas, Utah, and New Jersey) experienced enough growth in the use of MOUD among individuals referred to treatment by the criminal justice system to achieve at least 50% of individuals receiving MOUD in criminal justice–referred OUD treatment in 2021. The persistence in the disparity across many states suggests differences between OUD treatment that originates in the criminal justice setting and other settings remain significant in many states, and that it will require further efforts to address. Potential causes could include stigma and preference for nonmedication treatment by officials in the criminal justice system,^32^ individual and family stigma around MOUD among individuals with criminal justice system involvement,^33^ fear of forced withdrawal from MOUD,^33^ differences in the kinds of health care professionals willing to develop relationships with the criminal justice system,^33,34^ and institutional barriers to availability of MOUD in criminal justice settings.^33^

Future research should tease out mechanisms for the continued disparity and targets for intervention by collecting and analyzing data on the preferences and decisions of criminal justice officials and entities. Studies are also needed to examine the causal effects of initiatives to boost MOUD use in criminal justice settings, such as recently approved and pending state Medicaid waivers to waive the federal inmate exclusion policy,^35^ on health and public safety outcomes. These efforts will inform interventions to address the poor outcomes experienced by individuals with criminal justice system involvement.

### Limitations

The study had several limitations. First, quality assurance of TEDS-A data at the federal level is limited, leading to differences in reporting across states. To our knowledge, validation of how accurately TEDS-A data measure MOUD use has not been conducted. To the best of our ability, we addressed this by excluding states with inadequate data reporting and by comparing differences in the probabilities that admissions included MOUD (rather than the overall number). Nevertheless, poor reporting may still lead us to underestimate or overestimate the extent of MOUD use in some areas. Second, the TEDS-A data only include admissions to specialty substance use treatment facilities. Thus, we were unable to examine OUD treatment episodes that involve health care professionals who practice outside these settings (eg, in primary care). Other research, however, suggests this is not a large issue in the context of our study because primary care clinicians are less likely to receive referrals from the criminal justice system relative to specialty substance use treatment facilities.^34^

Third, we were unable to distinguish which medications (buprenorphine, methadone, or naltrexone) contributed to reduced disparities in MOUD use for individuals with and without criminal justice system involvement. Recent press investigations suggest that naltrexone has been heavily marketed in carceral settings,^36^ despite buprenorphine and methadone being more established medications for reducing overdose risk. Higher use of naltrexone in carceral settings could lead us to underestimate disparities in effective medication use; however, other data suggest that naltrexone accounts for a small and declining share of MOUD use in carceral settings.^37^ Fourth, the TEDS-A data do not report detailed information on the types of substance use disorder treatment facilities that individuals are admitted to. This prevented us from being able to assess whether certain types of facilities (eg, opioid treatment programs) achieve better outcomes. Finally, unobservable changes (eg, in OUD severity) among the populations of individuals referred to treatment by the criminal justice system and other referral sources may have also contributed to changes in the probability of MOUD use over time, creating potential bias in our estimates of the extent to which disparities have been reduced.

## Conclusions

Despite modest improvements in the use of evidence-based treatment for OUD in criminal justice settings, clients with criminal justice involvement remain far less likely to receive evidence-based care than clients referred to treatment by other sources. The persistence of this problem across time and states highlights that additional efforts are urgently needed, particularly given the escalation of opioid overdose death rates in recent years. Improving tracking of the type and extent of medication use in criminal justice referred treatment (eg, through improving standards and oversight of TEDS data reporting) is also needed for ongoing assessments of how well policy is doing with respect to providing evidence-based care to the highly vulnerable population of individuals with OUD and criminal justice system involvement.

## Notes

Supplement 1. eAppendix 1. Estimating Equations eAppendix 2. Sensitivity Analysis with Additional States eTable 1. Sample exclusions to the TEDS-A data eTable 2. Primary substances involved in opioid admissions over time eTable 3. Sources of referrals to treatment from the criminal justice system eTable 4. Predicted probability of medication for opioid use disorder use during treatment by referral source, 2014-2021 eTable 5. Sensitivity analysis for Table 2 with additional states eTable 6. Results from state-level disparities analysis eTable 7. Differential trend in the probability of MOUD use in criminal legal-referred treatment by state eFigure 1. Sensitivity analysis of Figure 1 with additional states. eReference

Supplement 2. Data sharing statement
