# Supplementary material for: Disparities in Medication Use for Criminal Justice System–Referred Opioid Use Disorder Treatment
Source: JAMA Health Forum. 2024 Sep 6;5(9):e242807. doi: 10.1001/jamahealthforum.2024.2807 (PMC11380100; doi:10.1001/jamahealthforum.2024.2807)
Supplement: Supplement 1. — eAppendix 1. Estimating Equations eAppendix 2. Sensitivity Analysis with Additional States eTable 1. Sample exclusions to the TEDS-A data eTable 2. Primary substances involved in opioid admissions over time eTable 3. Sources of referrals to treatment from the criminal justice system eTable 4. Predicted probability of medication for opioid use disorder use during treatment by referral source, 2014-2021 eTable 5. Sensitivity analysis for Table 2 with additional states eTable 6. Results from state-level disparities analysis eTable 7. Differential trend in the probability of MOUD use in criminal legal-referred treatment by state eFigure 1. Sensitivity analysis of Figure 1 with additional states. eReference [file jamahealthforum-e242807-s001.pdf]

# Supplemental Online Content

Donahoe JT, Donohue JM, Saloner BK. Disparities in medication use for criminal justice system–referred opioid use disorder treatment. *JAMA Health Forum*. 2024:5(9):e242807. doi:10.1001/jamahealthforum.2024.2807

eAppendix 1. Estimating Equations

eAppendix 2. Sensitivity Analysis with Additional States

eTable 1. Sample exclusions to the TEDS-A data

eTable 2. Primary substances involved in opioid admissions over time

eTable 3. Sources of referrals to treatment from the criminal justice system

eTable 4. Predicted probability of medication for opioid use disorder use during treatment by referral source, 2014-2021

eTable 5. Sensitivity analysis for Table 2 with additional states

eTable 6. Results from state-level disparities analysis

eTable 7. Differential trend in the probability of MOUD use in criminal legal-referred treatment by state

eFigure 1. Sensitivity analysis of Figure 1 with additional states.

eReference

This supplemental material has been provided by the authors to give readers additional information about their work.

© 2024 Donahoe JT et al. *JAMA Health Forum.*

For our primary analysis, we estimated regressions of the following form,

$$E\left[Y_{i,t}\middle|C_{it},X_{it}\right] = F\left(\beta_0 + \beta_1 C_{i,t} + \beta_2 T_t + \beta_3 C_{i,t} \times T_t + X'_{i,t}\gamma\right), \tag{1}$$

with

- $Y_{i,t}$ = indicates whether admission $i$ at time $t$ included medication for opioid use disorder (MOUD);
- $C_{i,t}$ = indicates whether treatment was referred by the criminal legal system;
- $T_t$ = a linear trend for year;
- $C_{i,t} \times T_t$ = an interaction between criminal legal referrals and year; and
- $X_{i,t}$ = a vector of individual-level covariates (age, sex, race and ethnicity, educational attainment, co-occurring substance use, and Census region).

The functional form $F(\cdot)$ was assumed to be logistic.

**Table 1** reports average marginal effects from equation (1). Following prior literature, we test the interaction between criminal legal referrals to treatment and time using the cross-partial derivative of equation (1) with respect to criminal legal referrals and time.[1]

For **Figure 1**, we estimated several parameters from equation (1). The first two were the differential trends in the probability of MOUD use for individuals with and without criminal legal referrals to treatment. These are given by the following equations:

$$\sum_{i=1}^{N} \frac{\partial F}{\partial v} \hat{\beta}_2 \tag{2}$$

and

$$\sum_{i=1}^{N} \frac{\partial F}{\partial v} \left(\hat{\beta}_2 + \hat{\beta}_3\right), \tag{3}$$

where equation (2) estimates the average time-trend for non-criminal legal referred admissions and equation (3) estimates the average time-trend for criminal legal referred admissions.

Next, we estimated predicted probabilities of MOUD use for individuals with and without criminal legal referrals to treatment each year that were adjusted for patient characteristics. Specifically, using estimates of equation (1), we estimated the predicted probability of MOUD use under counterfactuals that reassigned all admissions to a given year (e.g., 2014) and type of referral (e.g., criminal legal or other). Results are presented in **Figure 1** and corresponding numerical results are reported **eTable 4**.

In **Figure 1**, we also report the adjusted difference in the probability of MOUD use for individuals with and without criminal legal referrals to treatment in 2014 (the beginning of our time series) and 2021 (our most recent year of data).

In **Figure 2**, we stratified equation (1) by state. Using these estimates, we report separate time-trends in the probability of MOUD use (and 95% confidence intervals) for individuals with criminal legal referrals to treatment in each state. We also compare these trends to the trends that were needed for half of individuals with criminal legal referred treatment to receive MOUD in 2021 (approximately the probability that non-criminal legal referred admissions received MOUD in 2021). Specifically, this was calculated as the annualized difference in each state's predicted probability of MOUD among criminal legal referred individuals in 2014 (from the state stratified regressions) and 50%. As an example, in 2014, 26.1 percent of criminal legal-referred treatment admissions in Arkansas included MOUD. To have achieved a 50 percent probability by 2021, growth in the probability of MOUD use among criminal legal-referred treatment admissions would have had to have been (50-26.1)/(2021-2014)=3.41 percentage points per year.

Numerical values for each state are reported in **eTable 5**.

© 2024 Donahoe JT et al. JAMA Health Forum.

eAppendix 2. Sensitivity Analysis with Additional States

In this section we conduct a sensitivity analysis and replicate **Tables 2** and **Figure 1** with additional states, by restricting the data to 2014 and 2021 and including 44 states and the District of Colombia which have data in those two years and report data for the MOUD variable. We continue to exclude 5 states that do not report any data in either 2014 or 2021 (Delaware, Idaho, Oregon, South Carolina, and Washington) and one that reports missing for the MOUD variable for all admissions (West Virginia). We modify our empirical approach by replacing the continuous variable for year in equation (1) with a binary indicator for 2021. Results are presented below (**eTable 5** and **eFigure 1**) and implications are very similar compared to the main estimates in our paper. In particular, they also show the following facts: (1) use of MOUD was lower for criminal legal-referred treatment in all years; (2) use of MOUD increased overall; (3) use of MOUD increased differentially more for individuals in criminal legal-referred treatment, helping to reduce the disparity; and (4) the rate of increase in MOUD use in criminal legal-referred treatment was not sufficient to close the disparity and a persistent disparity in MOUD use between individuals referred to treatment by the criminal legal system and others remained in 2021. The numerical estimates are also very similar: e.g., in 2021, the main analysis estimates a disparity of 15.6 percentage point in the probability of MOUD use between individuals in criminal legal-referred treatment and others. The sensitivity analysis with additional states estimates an even larger disparity of 17.7 percentage points.

Overall, this sensitivity analysis shows that our conclusions about there being progress with respect to MOUD use in criminal legal settings, but persistent disparities, are not particularly sensitive to the exclusion of particular states for not reporting data or submitting data with inadequate quality during certain years.

© 2024 Donahoe JT et al. JAMA Health Forum.

## eTable 1. Sample exclusions to the TEDS-A data.

| | N |
|---|---|
| **Total admissions, 2014-2021** | 13,939,542 |
| Restricting to adults admissions with opioids as primary substance | 4,349,964 |
| Excluding states with inadequate data reporting | 3,650,520 |
| Excluding admissions with missing data | 3,235,445 |
| **Final analytic sample** | **3,235,445** |

*Notes.* Data are from the Treatment Episodes Dataset-Admissions (TEDS-A), 2014 to 2021.

© 2024 Donahoe JT et al. JAMA Health Forum.

## eTable 2. Primary substances involved in opioid admissions over time.

| Year | No. (%) | | |
|---|---|---|---|
| | Heroin | Non-prescription methadone | Other opiates and synthetics |
| 2014 | 333,988 (73.3) | 4,062 (0.89) | 117,723 (25.8) |
| 2015 | 361,813 (75.7) | 3,685 (0.77) | 112,341 (23.5) |
| 2016 | 383,882 (76.5) | 3,190 (0.64) | 114,786 (22.9) |
| 2017 | 403,568 (77.6) | 3,116 (0.60) | 113,594 (21.8) |
| 2018 | 413,504 (77.1) | 2,819 (0.53) | 120,001 (22.3) |
| 2019 | 362,651 (78.2) | 2,327 (0.50) | 98,906 (21.3) |
| 2020 | 278,303 (77.1) | 1,675 (0.46) | 80,938 (22.4) |
| 2021 | 236,900 (71.0) | 1,337 (0.40) | 95,411 (28.6) |

*Notes.* Data are from the Treatment Episodes Datset-Admissions (TEDS-A), 2014 to 2021.

© 2024 Donahoe JT et al. JAMA Health Forum.

## eTable 3. Sources of referrals to treatment from the criminal justice system.

| | No. (%) |
|---|---|
| **Source of referral** | |
| Probation/parole officer | 135,724 (26.6) |
| State/federal court | 84,638 (16.6) |
| Diversion program | 32,655 (6.4) |
| Other court | 31,720 (6.2) |
| Other legal entity | 23,662 (4.6) |
| Prison | 18,320 (3.6) |
| DUI/DWI | 6,836 (1.3) |
| Other | 39,575 (7.8) |
| Missing/unknown/not collected/invalid | 136,635 (26.8) |

*Notes.* Data are from the Treatment Episodes Datset-Admissions (TEDS-A), 2014 to 2021, among all clients referred to treatment primarily for opioids by the criminal legal system.

© 2024 Donahoe JT et al. JAMA Health Forum.

**eTable 4. Predicted probability of medication for opioid use disorder use during treatment by referral source, 2014-2021.**

| | Predictive margin [95% CI] |
|---|---|
| **Not criminal legal-referred** | |
| 2014 | 31.7 [31.62, 31.80] |
| 2015 | 34.1 [33.99, 34.14] |
| 2016 | 36.5 [36.42, 36.55] |
| 2017 | 39.0 [38.91, 39.02] |
| 2018 | 41.5 [41.44, 41.56] |
| 2019 | 44.1 [44.00, 44.15] |
| 2020 | 46.7 [46.58, 47.77] |
| 2021 | 49.3 [49.17, 49.40] |
| **Criminal legal-referred** | |
| 2014 | 8.8 [8.67, 8.92] |
| 2015 | 10.9 [10.82, 11.06] |
| 2016 | 13.5 [13.39, 13.63] |
| 2017 | 16.6 [16.44, 16.66] |
| 2018 | 20.1 [19.97, 20.21] |
| 2019 | 24.1 [23.98, 24.30] |
| 2020 | 28.7 [28.45, 28.89] |
| 2021 | 33.6 [33.34, 33.94] |
| N | 3,235,445 |

*Notes.* Reports predictive margins from estimates of equation (1) and data on opioid treatment admissions from the Treatment Episodes Dataset Admissions (TEDS-A), 2014-2021.

© 2024 Donahoe JT et al. JAMA Health Forum.

## eTable 5. Sensitivity analysis for Table 2 with additional states.

| | Average marginal effect (pp) (robust standard error) | [95% CI] | P-value |
|---|---|---|---|
| **Criminal legal referred** | -21.21<br>(0.13) | [-21.46, 20.96] | <0.001 |
| **Year = 2021** | 17.40<br>(0.11) | [17.18, 17.62] | <0.001 |
| **Criminal legal referred × year = 2021** | 6.51<br>(0.28) | [5.96, 7.05] | <0.001 |
| **Age (rel. 18-24)** | | | |
| Age 25-34 | 7.41<br>(0.15) | [7.11, 7.71] | <0.001 |
| Age 35-54 | 14.30<br>(0.16) | [13.98, 14.61] | <0.001 |
| Age 55+ | 22.37<br>(0.24) | [21.90, 22.84] | <0.001 |
| **Female** | 4.66<br>(0.11) | [4.45, 4.87] | <0.001 |
| **Race and ethnicity (rel. non-Hispanic White)** | | | |
| Hispanic | -0.16<br>(0.15) | [-0.45, 0.13] | 0.276 |
| Non-Hispanic American Indian/Alaskan Native | 3.92<br>(0.47) | [3.00, 4.84] | <0.001 |
| Non-Hispanic Black | 2.06<br>(0.17) | [1.73, 2.40] | <0.001 |
| Other race and ethnicity* | -2.97<br>(0.15) | [-3.52, -2.42] | <0.001 |
| **Education (rel. < high school)** | | | |
| High-school grad | -2.17<br>(0.12) | [-2.41, -1.93] | <0.001 |
| College grad | -5.72<br>(0.26) | [-6.23, -5.21] | <0.001 |
| **Primary opioid used (rel. heroin)** | | | |
| Non-prescription methadone | 14.11<br>(0.66) | [12.82, 15.39] | <0.001 |
| Other opiates and synthetics | -4.01<br>(0.12) | [-4.29, -3.77] | <0.001 |
| **Reported harmful alcohol use** | -12.04<br>(0.14) | [-12.31, -11.77] | <0.001 |
| **Reported harmful benzodiazepine use** | -8.23<br>(0.17) | [-8.56, -7.90] | <0.001 |
| **Census region (rel. Northeast)** | | | |
| Midwest | 2.94<br>(0.15) | [2.64, 3.24] | <0.001 |
| South | -18.13<br>(0.13) | [-18.39, -17.87] | <0.001 |
| West | 15.04<br>(0.16) | [14.73, 15.35] | <0.001 |
| Unadjusted mean | 35.22 | | |
| N | 750,093 | | |

*Notes.* Data are from the Treatment Episodes Dataset-Admissions (TESD-A), 2014 and 2021. The table reports average marginal effects from a logistic regression of the probability that treatment admissions involved use of medication for opioid use disorder (includes buprenorphine, methadone, and naltrexone) on each variable in the table. Robust standard errors are reported in parentheses. 95% confidence intervals are reported in brackets. * The other race and ethnicity category includes non-Hispanic individuals whose race was Asian, Native Hawaiian, other Pacific Islander, another single race, two or more races, or missing in the TEDS-A data.

© 2024 Donahoe JT et al. JAMA Health Forum.

## eTable 6. Results from state-level disparities analysis.

| State name | Probability of MOUD use (pp) (criminal legal-referred treatment) 2014 | Trend (95% CI) | Benchmark |
|---|---|---|---|
| Arkansas | 26.1 | -3.0 (-3.70, -2.31) | 3.41 |
| Washington D.C. | 13.5 | -0.8 (-2.09, 0.48) | 5.22 |
| Nebraska | 11.3 | -0.6 (-1.79, 0.59) | 5.54 |
| Colorado | 7.0 | -0.3 (-0.46, -0.17) | 6.15 |
| Missouri | 5.6 | -0.1 (-0.32, 0.07) | 6.34 |
| Louisiana | 3.0 | -0.1 (-0.36, 0.22) | 6.71 |
| Tennessee | 0.3 | 0.0 (-0.06, 0.01) | 7.10 |
| Hawaii | 0.6 | 0.0 (-0.23, 0.27) | 7.06 |
| Illinois | 3.0 | 0.1 (-0.14, 0.24) | 6.71 |
| California | 4.6 | 0.1 (-0.03, 0.19) | 6.48 |
| Wyoming | 2.4 | 0.2 (-0.07, 0.56) | 6.81 |
| North Carolina | 0.3 | 0.3 (0.21, 0.34) | 7.10 |
| South Dakota | 6.5 | 1.0 (0.22, 1.72) | 6.21 |
| Wisconsin | 0.7 | 1.1 (0.89, 1.29) | 7.04 |
| Alabama | 2.4 | 1.1 (0.71, 1.47) | 6.79 |
| Pennsylvania | 10.8 | 1.6 (1.33, 1.82) | 5.60 |
| Ohio | 15.0 | 1.7 (1.50, 1.92) | 5.00 |
| Texas | 1.5 | 1.8 (1.41, 2.13) | 6.92 |
| New Hampshire | 3.6 | 1.8 (1.26, 2.32) | 6.63 |
| Rhode Island | 14.5 | 1.9 (1.23, 2.67) | 5.07 |
| Maine | 26.8 | 2.1 (1.38, 2.77) | 3.31 |
| Arizona | 5.6 | 2.2 (1.39, 3.00) | 6.35 |
| Florida | 3.0 | 2.3 (1.89, 2.75) | 6.72 |
| Nevada | 0.9 | 2.4 (2.01, 2.73) | 7.01 |
| Kansas | 0.1 | 2.4 (1.71, 3.08) | 7.13 |
| Georgia | 1.2 | 3.1 (2.33, 3.86) | 6.98 |
| Michigan | 13.5 | 3.1 (2.86, 3.41) | 5.21 |
| Iowa | 3.2 | 3.2 (2.62, 3.79) | 6.68 |
| Kentucky | 9.1 | 3.3 (3.04, 3.55) | 5.84 |
| North Dakota | 0.0 | 3.3 (2.18, 4.52) | 7.14 |
| Connecticut | 4.0 | 3.7 (3.45, 3.87) | 6.57 |
| Indiana | 2.6 | 4.0 (3.67, 4.35) | 6.76 |
| Mississippi | 0.1 | 4.2 (3.11, 5.24) | 7.13 |
| Minnesota | 12.5 | 4.3 (4.01, 4.64) | 5.35 |
| Massachusetts | 6.7 | 6.0 (5.74, 6.18) | 6.18 |
| Vermont | 22.0 | 6.1 (5.44, 6.72) | 3.99 |
| New York | 16.7 | 6.4 (6.30, 6.53) | 4.76 |
| Alaska | 2.0 | 7.2 (6.24, 8.07) | 6.85 |
| Utah | 1.6 | 7.4 (7.05, 7.72) | 6.92 |
| New Jersey | 3.8 | 9.3 (9.20, 9.47) | 6.60 |

*Notes.* Data are from the Treatment Episodes Dataset Admissions (TEDS-A), 2014-21. Results report the predicted probability of MOUD use among criminal legal-referred treatment admissions in each state in 2014, the trend in the probability of MOUD use among criminal legal-referred treatment admissions from 2014-2021 (from the logistic regression models described in Supplementary Appendix Section I), and what the trend would have needed to be to achieve 50 percent probability of MOUD use among individuals with criminal legal referrals to treatment in 2021 (approximately the probability of MOUD use among individuals without criminal legal referrals to treatment).

© 2024 Donahoe JT et al. JAMA Health Forum.

## eTable 7. Differential trend in the probability of MOUD use in criminal legal-referred treatment by state.

| State name | Criminal legal-referred × year (AME) | (95% CI) | p-value |
|---|---|---|---|
| Arkansas | -3.26% | (-4.08, -2.44) | <0.001 |
| Washington D.C. | -0.13% | (-1.46, 1.20) | 0.849 |
| Nebraska | -0.59% | (-2.09, 0.91) | 0.442 |
| Colorado | -3.96% | (-4.18, -3.75) | <0.001 |
| Missouri | -0.68% | (-0.94, -0.42) | <0.001 |
| Louisiana | 0.17% | (-0.15, 0.49) | 0.304 |
| Tennessee | -0.04% | (-0.09, 0.001) | 0.056 |
| Hawaii | 1.04% | (0.33, 1.75) | 0.004 |
| Illinois | 1.32% | (1.09, 1.56) | <0.001 |
| California | 1.68% | (1.55, 1.81) | <0.001 |
| Wyoming | -0.44% | (-1.04, 0.15) | 0.141 |
| North Carolina | -3.08% | (-3.20, -2.96) | <0.001 |
| South Dakota | -2.18% | (-3.21, -1.15) | <0.001 |
| Wisconsin | -2.51% | (-2.81, -2.21) | <0.001 |
| Alabama | -0.36% | (-0.85, 0.13) | 0.147 |
| Pennsylvania | -1.02% | (-1.31, -0.73) | <0.001 |
| Ohio | 1.00% | (-0.73, -1.26) | <0.001 |
| Texas | -2.47% | (-2.85, -2.09) | <0.001 |
| New Hampshire | -0.63% | (-1.27, -0.01) | 0.054 |
| Rhode Island | -1.78% | (-2.53, -1.03) | <0.001 |
| Maine | 2.54% | (-1.80, -3.28) | <0.001 |
| Arizona | 0.10% | (-0.80, 0.99) | 0.829 |
| Florida | -1.52% | (-1.98, -1.06) | <0.001 |
| Nevada | -0.13% | (-0.84, 0.58) | 0.716 |
| Kansas | -1.87% | (-2.70, -1.03) | <0.001 |
| Georgia | 1.83% | (-0.95, -2.72) | <0.001 |
| Michigan | 0.70% | (-0.41, -0.99) | <0.001 |
| Iowa | -1.39% | (-2.06, -0.71) | <0.001 |
| Kentucky | 1.29% | (-0.99, -1.59) | <0.001 |
| North Dakota | -0.78% | (-2.09, 0.54) | 0.247 |
| Connecticut | 3.26% | (-3.03, -3.50) | <0.001 |
| Indiana | -2.75% | (-3.21, -2.29) | <0.001 |
| Mississippi | -0.66% | (-1.83, 0.51) | 0.271 |
| Minnesota | 2.67% | (-2.32, -3.02) | <0.001 |
| Massachusetts | 4.28% | (-4.05, -4.52) | <0.001 |
| Vermont | 1.11% | (-0.41, -1.80) | 0.002 |
| New York | 2.14% | (-2.01, -2.26) | <0.001 |
| Alaska | 0.17% | (-0.83, 1.18) | 0.736 |
| Utah | 2.96% | (-2.49, -3.42) | <0.001 |
| New Jersey | 3.89% | (-3.73, -4.04) | <0.001 |

*Notes.* Data are from the Treatment Episodes Dataset Admissions (TEDS-A), 2014-21. Results report the average marginal effect of the interaction between criminal-legal referred treatment and year from the logistic regression models described in **Supplementary Appendix Section I**, separately for each state.

© 2024 Donahoe JT et al. JAMA Health Forum.

**eFigure 1. Sensitivity analysis of Figure 1 with additional states.**

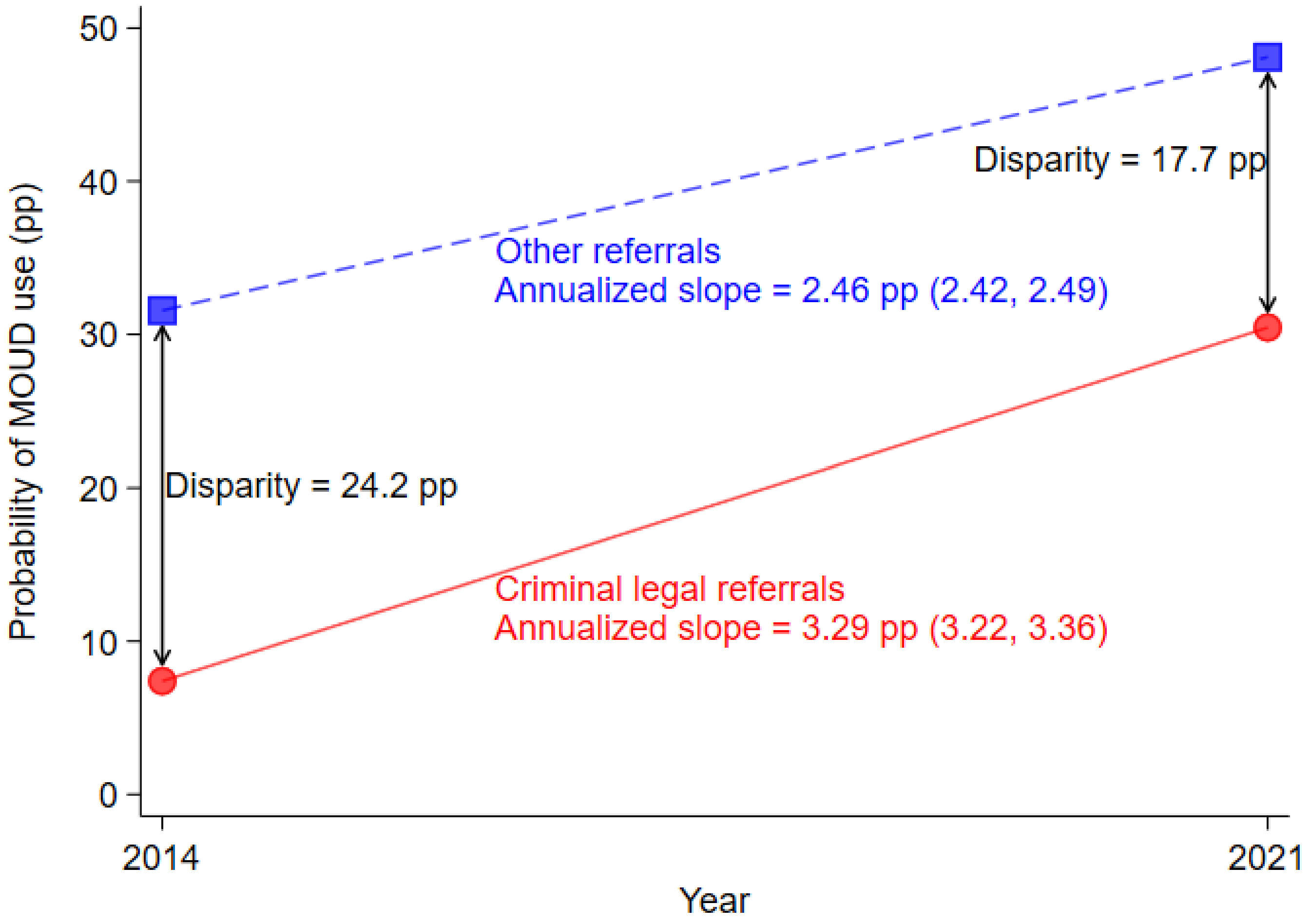


*Notes.* Data are from the Treatment Episodes Dataset-Admissions (TESD-A), 2014 and 2021. The markers denote the probability that a treatment admission included medication for opioid use disorder (MOUD) in each year, separately for clients referred to treatment by the criminal legal system (depicted as circles) and others (depicted as squares), adjusted for patient characteristics. The figure overlays annualized trend lines for each type of treatment referral (the difference between 2021 and 2014, divided by the number of years between). The slopes (and 95% confidence intervals) for each annualized time trend are reported below each line in the figure. The figure also reports the disparity in the probability of MOUD use for individuals referred to treatment by the criminal legal system and others (holding individual-level characteristics fixed) in 2014 and 2021.

© 2024 Donahoe JT et al. JAMA Health Forum.

eReference

1. Karaca-Mandic, P., Norton, E. C. & Dowd, B. Interaction Terms in Nonlinear Models. *Health Serv. Res.* **47**, 255–274 (2012).

© 2024 Donahoe JT et al. JAMA Health Forum.
