# Supplementary material for: Disparities in Medication Use for Criminal Justice System–Referred Opioid Use Disorder Treatment
Source: JAMA Health Forum. 2024 Sep 6;5(9):e242807. doi: 10.1001/jamahealthforum.2024.2807 (PMC11380100; doi:10.1001/jamahealthforum.2024.2807)
Supplement: Supplement 2. — Data sharing statement [file jamahealthforum-e242807-s002.pdf]

Data Sharing Statement

Donahoe. Disparities in Medication Use for Criminal Justice System–Referred Opioid Use Disorder Treatment. *JAMA Health Forum*. Published September 06, 2024. doi:10.1001/jamahealthforum.2024.2807

Data
Data available: Yes
Data types: Deidentified participant data
How to access data: Data are publicly available at: https://www.datafiles.samhsa.gov/dataset/treatment-episode-data-set-admissions-2000-2021-teds-2000-2021-ds0001.
When available: With publication

Supporting Documents
Document types: Statistical/analytic code
How to access documents: Code will be made publicly available via supplementary materials to the publication.
When available: With publication

Additional Information
Who can access the data: Anyone requesting the data.
Types of analyses: Any type of analysis.
Mechanisms of data availability: Any mechanism.
